# Benefits and Limitations of Teledermatology in German Correctional Facilities: Cross-Sectional Analysis

**DOI:** 10.2196/58712

**Published:** 2025-05-07

**Authors:** Brigitte Stephan, Kathrin Gehrdau, Christina Sorbe, Matthias Augustin, Martin Scherer, Anne Kis

**Affiliations:** 1 Institute for Health Services Research in Dermatology and Nursing, University Medical Center Hamburg-Eppendorf, Martinistraße 52, Hamburg, 20246, Germany, 49 40741055428; 2 A+ Videoclinic GmbH, Munich, Germany; 3 Institute for General Medicine, University Medical Center Hamburg-Eppendorf, Hamburg, Germany

**Keywords:** dermatology, dermatologist, dermatological, dermatological care, accessibility, prison health, teledermatology, telemedicine, telehealth, virtual health, virtual medicine, remote consultation, telephone consultation, video consultation

## Abstract

**Background:**

Teledermatology consultations offer the advantage of rapid diagnosis and care. Since 2019, our institute at the University Medical Center Hamburg-Eppendorf has been part of an interdisciplinary team for teledermatology support in German prisons as an alternative to extramural transports of patients.

**Objective:**

This study aims to analyze the benefits and limitations of teledermatology for patients with limited access to medical specialties.

**Methods:**

We conducted a descriptive cross-sectional analysis of 651 teleconsultations from prisons from February 2020 to April 2023. All cases were performed in a store-and-forward (asynchronous mode) and optional hybrid live (synchronous) consultation for the patient or in-house staff.

**Results:**

The main advantage of this case processing was the avoidance of external transport. Of the 651 teleconsultations, 608 (93.4%) could be finalized with telemedical support and 43 (6.6%) required additional workup, including verifications of the type of tumors (n=22, 51%), which needed biopsies, and open cases that were inflammatory (n=11, 26%) or involved infectious skin conditions (n=5, 12%). Digital imaging of the skin lesions improved with the experience of the personnel but remained a challenge, with the photo quality depending on the technical devices or available broadband supply.

**Conclusions:**

Hybrid teledermatology consultation represents an effective and resource-saving method of providing specialized care to patients in situations with limited access to medical specialties. The video consultations with experts and exchange of knowledge about the cases presented opened the opportunity to support and train intramural colleagues. One of the main challenges remains the quality of digital imaging and transmission.

## Introduction

The international recommendations for medical care in correctional facilities set high standards [1]. According to global agreements stated by the World Health Organization, prisoners should receive equivalent medical care in comparison to the standards for the respective population [2,3]; this includes primary care as well as access to all medical specialties, which is challenging for the correctional system [4-7].

### Need for Health Services in Correctional Facilities

On average, prisoners are younger compared to the normal population, and more than 90% are male, which indicates a different scope of medical issues [8]. However, prisoners also have a higher proportion of individuals with chronic diseases, especially mental disorders or chronic infections like hepatitis, requiring ongoing medical care [9-14].

Prisons in Germany ensure these medical services not only using in-house resources but also regularly using external medical service providers to fulfill these tasks [15-17]. For dermatology issues, this means complete reliance on external dermatology facilities because none of the more than 280 correctional facilities in Germany provide in-house solutions.

### Economic Aspects of Medical Care in Prisons

Following the principle of equal standard of medical care for prisoners as people living in the community, the scope of medical services in German correctional institutions should correspond to the scope of services provided by the statutory health insurance funds. However, prisoners are no longer covered by statutory health insurance when they start their prison sentence in Germany [7]. The costs for medical care must be covered by the prison authorities of the federal state. The expenses for the correctional facilities [18,19] as well as the medical care in the German correctional system have risen steadily, and the federal states of Germany have to cover these costs [20]. A substantial amount is caused by personnel and transport costs. New solutions are needed to both provide the required standards of care and control costs [21].

### Use of Teledermatology for Correctional Facilities in Germany

During the last decades, telemedicine in various settings has become a useful tool to support health services [22]. In 2018, the 121st German Medical Congress approved an amendment to the medical professional code of conduct (Muster-Berufsordnung für die in Deutschland tätigen Ärztinnen und Ärzte [MBO-Ä]) and enabled remote telemedical care [23]. Since 2019, German correctional institutions have started to use telemedicine for dermatology requests [20,24]. Initially, this service was provided through a pilot project by the Ministry of Justice of Baden-Württemberg and expanded to other German federal states and correctional facilities in subsequent years. Currently, these dermatology teleconsultations are offered on a fixed weekly basis for acute cases on demand within 24 hours. The prison can choose between a store-and-forward mode (asynchronous operation) with photos and a standardized form only or an additional hybrid option with a live video consultation in an interdisciplinary round including the treating primary physician simultaneously (synchronous operation). This allows a direct exchange of medical information as well as the opportunity for further counseling with colleagues in charge of the case. Both modes represent an alternative to transportation and presentation of prisoners in dermatology facilities outside the correctional institution, and we experience an increasing number of requests to our institute from German correctional institutions.

Although the rate of completed cases through video consultations in either store-and-forward mode or in live presentation is high [8], there are limitations to the processing of certain requests. With this paper, we analyzed 651 cases of our teleconsultations for German correctional facilities from February 2020 to April 2023. This study aimed to provide evidence on the value of teledermatology care provided to German prisons and detect limitations for this mode of service with a focus on cases that could not be processed without live on-site consultations.

## Methods

### Overview

A systematic comparison of the advantages and disadvantages of video consultations for prisoners was carried out based on a descriptive cross-sectional analysis of 651 applications for teleconsultations in German correctional facilities from February 2020 to April 2023, supplemented with literature research. The selection of the evaluation criteria was based on quality criteria from the German guidelines for teledermatology from 2020 [25].

We retrieved all data from the documented request forms from February 2020 until April 2023. Data included:

- clinical symptoms and anamnestic information of skin disease;
- patients’ demographic data (age and gender);
- preliminary diagnosis and questions from the in-house medical team of the prison regarding the case (eg, special issues such as occupational or infectious matters, quarantine, or isolation);
- documentation of the consultation, including details of onset and clinical appearance of the skin disease, and number and quality of photos sent with the request; and
- further anamnestic information retrieved from the virtual presentation of the case and dermatological diagnosis and suggestions for treatment.

### Ethical Considerations

The ethics committee of the Medical Association of Hamburg confirmed that there was no further ethical approval necessary for retrospective analysis of anonymized data in accordance with the ethical standards of the responsible committees (institutional or regional) and with the Helsinki Declaration of 1975, as revised in 1983.

### Spatial Analyses

Part of this study was a spatial assessment of the potential physical accessibility of prisoners to dermatologists. The data basis for all 284 prison locations was a list from the justice portal of the federal and state governments from 2020. After geocoding and referencing the locations, they were loaded and mapped in a geographic information system in ArcGIS v. 10.8.1 (ESRI). As a proxy for accessibility, physician density was compared to correctional facility locations. Data were provided by Kassenaerztliche Bundesvereinigung (KBV; data status 2022).

### Statistical Analysis

We conducted a descriptive analysis of all variables with SPSS v.26 (IBM Corp), including demographic data (eg, age and gender) and medical data (eg, diagnosis, suggestions for therapy, and further management of the skin disease). Thereby, we categorized open entries, for example, therapy modalities, into categories such as topical or systemic therapy. We conducted a subgroup analysis for cases that were not closed after one video consultation regarding diagnoses or recommendations for further management modalities.

## Results

### Overview

We offer teledermatology support for correctional facilities in Germany. As of May 2023, a total of 38 prisons in 6 federal states in Germany used this service regularly, with rising numbers. Of the 651 cases analyzed, we were asked for 412 diagnoses, 497 therapy recommendations, and 62 follow-ups for reconsidering or changed skin conditions. We provided a time-saving response within 1 week after the request or, in urgent cases, a response within 24 hours in store-and-forward mode. The most frequently diagnosed skin diseases were eczema, skin infections, acne or acneiform dermatitis, and tumor differentiation [26].

### Accessibility of Dermatologists Through Telemedical Consultations: Spatial Considerations for Physical Contact

**Figure 1.**
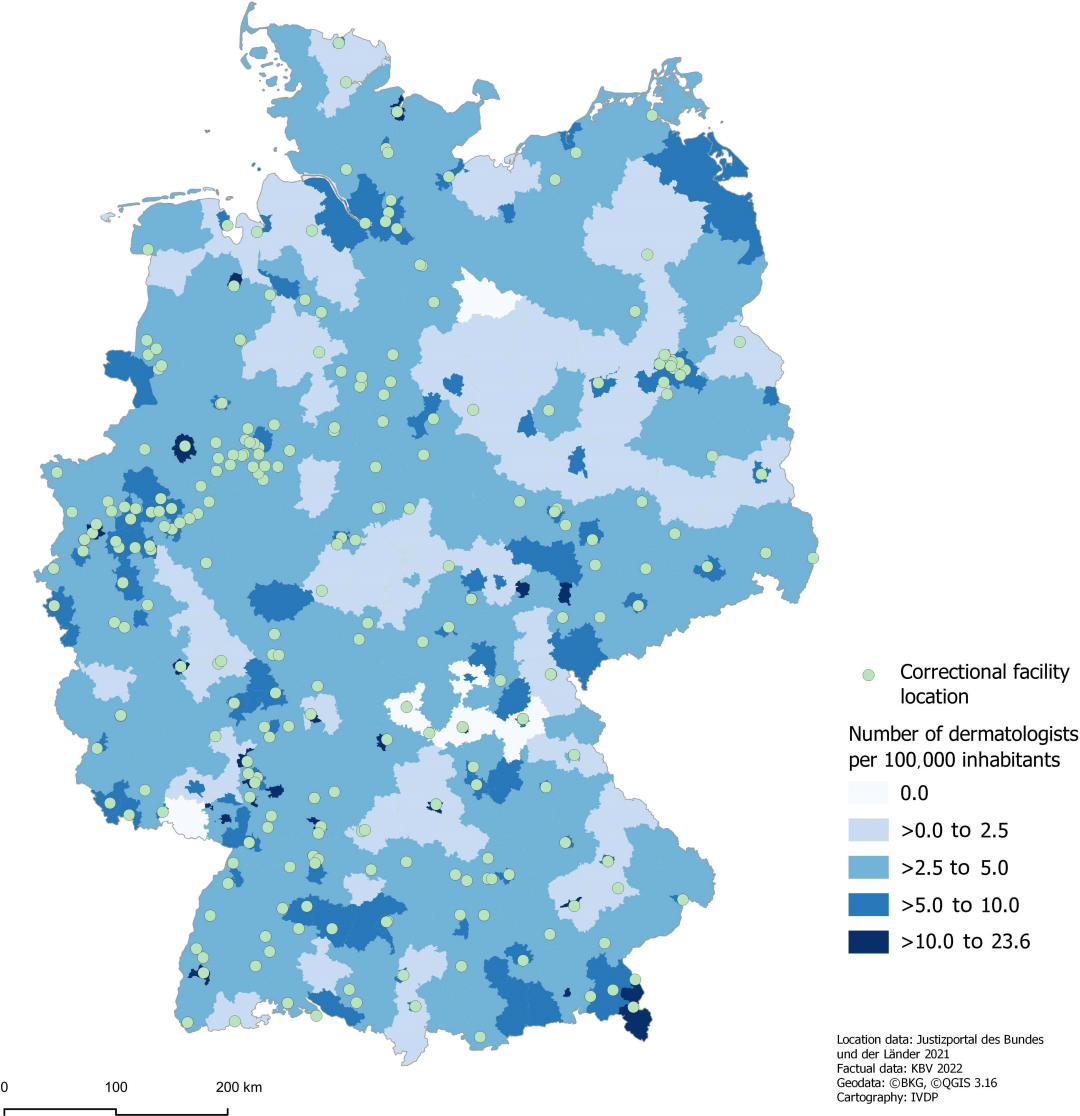
Cartographic representation of all prison locations in Germany. Cartographic representation of prisons in Germany (green dots) and the density of registered dermatologists per 100,000 inhabitants (blue color scheme). Only a few districts have a high density of dermatologists, between 10-23.6 per 100,000 inhabitants. The majority of districts have between 2.5-5.0 registered dermatologists or fewer, and most prisons are situated outside the metropolitan areas. This results in heterogeneous accessibility.

In Germany, there are 284 locations for correctional facilities distributed across all 16 federal states (Figure 1) [25]. The distribution of the prisons differs from being centrally located in metropolitan areas to being far from cities in rural areas. This is important for the accessibility and vicinity of medical specialists (eg, dermatologists).

### Avoidance of Transports Outside the Prison

The transport of an inmate to extramural facilities requires at least two staff members, and in cases of low personnel resources, the police forces have to support the institutional staff in Germany. We analyzed our 651 cases for finalization and closure of the case in terms of sufficient data for a diagnosis, recommended therapeutic options in-house, and a need for further diagnostic or therapeutic procedures requiring transport to outside facilities.

Of the 651 cases, 608 could be finalized with a diagnosis and recommendations for treatment and further management without need for extramural transportation of the patient, 83 had to send additional material like laboratory results or images for proceeding with the cases, and 43 could not be solved by teledermatology and consequently needed extramural support on site.

Of the 43 cases, 22 were questions concerned with the type of tumor (Table 1), for which we recommended live examination on site or biopsies that could not be performed inside the prison itself. Usually, the in-house physicians do not perform biopsies, and therefore, the teledermatology consultation did not lead to a successful closure of the case in these requests.

A total of 2 cases were exanthems of uncertain origin with recommendation for biopsies; 6 cases were eczema with missing anamnestic or clinical information for a differential diagnosis and proper conduct; 5 cases were suspected skin infections, and the facilities did not respond to our request for further clinical information or laboratory results like swabs or culture; 1 case showed the clinical picture of psoriasis but insufficient imaging of the extent and a lack of information about comorbidities and previous management; 2 cases sent images of acneiform lesions but lacked clinical information; 1 case asked for differential diagnosis of dermatitis herpetiformis Duhring but did not send sufficient clinical or anamnestic data; and 1 case asked for management of alopecia without further clinical or anamnestic information. Interestingly, the number of cases that were sent without a question for dermatology was rare (1 question for dysesthesias with a neurological background and 2 cases without any information about a skin problem).

According to the Guideline for Teledermatology [27], general screening for skin cancer should not be made by teledermatology alone. If the prison was able to send accurate dermoscopic images, there was an opportunity to provide a statement about the type of lesion or to rule out malignancies, but if the digital imaging was not sufficient, there was a need to consult the physician in charge on site. Cases with the need for general inspection of the entire body or detection of small pigmented lesions as well as skin conditions with the need for palpation (eg, lymph nodes), had to be referred to a physician on site if available or transported to outside facilities. Of our 651 cases, 96 asked for diagnosis of different types of tumors, of which 22 could not be solved by digital imaging, clinical description, and additional video consultation. Some cases could be solved by educating the in-house physician about screening procedures.

**Table 1.** Distribution of video consultations with closure based on uploaded request forms, photos sent, and live presentation online.

Case status	Cases (N=651), n
Solved cases	608
Unsolved cases	43
Tumors	22
Eczemas	6
Infections	5
Acneiform	2
Exanthemas	2
No information	2
Psoriasis	1
Nondermatological	1
Duhring	1
Alopecia	1

### Telemedical Expert Exchange of Knowledge With Intramural Colleagues of the Correctional Facilities

Due to the fact that no prison in Germany has dermatology resources, the intramural colleagues in charge of the facility service, mostly general practitioners, have to treat and follow dermatological cases. With the help of telemedical expertise and discussions online, we allowed for the exchange of knowledge about the case presented or skin diseases in general and, therefore, enabled the colleagues to become more capable of proceeding with further cases on their own. All of our video consultations were attended to by in-house medical staff from the prison, and in all virtual visits, we exchanged information about the present skin disease as well as therapeutic options and guidelines.

### Data Provision and Photo Documentation From Institution to Dermatologist

The number of photos per case was not limited, and 0-27 photos were sent for one request. Insufficient photo quality of at least one photo was found in 124 of the 651 requests from February 2020 to April 2023, but only 12 cases did not upload any sufficient imaging. The quality of the photos sent improved with the number of cases an individual prison performed and if the service provider supported technical solutions and devices.

## Discussion

This study aimed to identify the benefits and limitations of teledermatology support for prisons in Germany (Textbox 1). Prisons are mostly situated outside of the city centers without proximity to medical specialties like dermatologists. The consequence of long distances to the dermatology institutions is the impossibility of visits on site by the nearest dermatologists. Although in metropolitan areas the distances might be low, it does not necessarily mean the availability of dermatologists who are willing to visit one patient in a correctional facility, considering the difficult and time-consuming organization of access and visit schedules aside from their routine medical care and duties.

With the abovementioned German prison locations and physician density of all 16 federal states in mind, the willingness and reality of established specialists coming to the prison should be questioned [28]. The time and effort required of medical specialists due to the treatment of prisoners in prison could force physicians to reject the requests. Even though the medical specialists will be paid based on treatment and the medical fee schedule [29], the opportunity costs could be seen as too high. Therefore, it is not unusual for there to be a noticeable shortage of physicians in the prison system [15].

With our institute, we provided time-saving access to dermatology expertise and avoided transportation in most cases. Compared to general waiting times in Germany for specialist appointments, this is a significant benefit for the patients [30]. Transporting a prisoner to an off-site facility is a challenge to security and human resources. Consequently, for the prison and the community, the risk of an outbreak is very low if the medical specialist comes to the prison instead of bringing the prisoner to the physician [31]. Avoidance of transports outside the prison enhances security and saves resources. In cases with telemedical support, transport is no issue. A recent systematic review of the implementation of telemedicine found that public security and the reduction of the risk of a prisoner escaping are some of the main arguments to enable the successful implementation of telemedicine interventions [32]. From the perspective of prisons and the community, and probably for the prisoner as well, due to a lack of exposure in public, avoiding transport for medical needs with the alternative option of telemedical consultation is preferable. Furthermore, the additional hourly security and transportation costs for each external practice visit should be considered [33].

Although the rate of finalized cases was high (608/651 cases) [8], we could not finalize every case by teledermatology with hybrid store-and-forward information, digital imaging, and video consultation alone. For pigmented lesions, differentiation should not be performed with video consultation alone, and digital imaging could not provide sufficient photo quality in all cases. From 97 requests for tumors or pigmented lesions, a total of 244 photos (including dermoscopic imaging) with a maximum of 9 photos for one case were documented, but in 12 cases, no photo was sent, and in 2 cases, the photo quality was insufficient for a statement concerning type or diagnosis.

Looking at the analyzed cases, we saw a benefit in the interdisciplinary exchange of medical expertise, especially for chronic inflammatory skin diseases like atopic dermatitis or psoriasis where the potential change in clinical picture and severity of the disease poses sometimes challenging situations and the need for knowledge about modern therapeutic options and updated guidelines for therapeutic approaches. Especially for darker skin types who form a relevant proportion of patients in custody, it is helpful to have experience with the different appearance of skin diseases on dark skin, a knowledge which cannot be anticipated for intramural medical personnel. If we compare it to dermatology consultations of previous years, where we saw prisoners live in our outpatient clinic in Hamburg with transports to our institute from the surrounding prisons, we found that the accompanying nonmedical personnel were not involved in the patient’s case or disease and could not provide further information if needed. In reverse, the in-house physicians of the prison did not have direct contact with us and did not benefit from medical exchanges. Only a short medical letter provided the connection and flow of information between the prison and the dermatologist. In direct video consultation, the exchange of information from the medical file of the patient and information about the skin disease from the dermatologist was used for all cases discussed online, which was highly appreciated by both sides.

Not all prisons using teleconsultations were willing to send photos and dermoscopic imaging accompanying the request form due to data safety issues. Although the service provider offered a certified cloud solution, the individual prison responsible decided not to use this option. On the other hand, the resolution on both sides of the camera in live discussions online is not sufficient to detect high-resolution skin efflorescences such as telangiectasias, pigmented spots, or small appendages. This can be due either to the technology or to the available broadband supply. Studies show that, especially in rural areas, the conditions for sufficient broadband availability are not available everywhere to enable fast and, especially for medicine, reliable data transmissions [34].

Teleconsultations provide facilitated and time-saving access to medical specialties, and dermatology is most suitable for telemedical visualization of medical skin conditions. Both remote store-and-forward conduct as well as online discussion support the diagnosis of skin conditions and enable follow-up and guidance of therapy regimens.

Despite the significant progress telemedicine and teledermatology have made, the transition of medical treatment from face-to-face to only digital formats is not always appropriate to meet medical goals [35]. Overall, prisons have to analyze their demand for any kind of medical service that cannot be covered by their in-house general practitioner. The skin disease in focus, the possibilities of digital imaging of the condition, interventions needed, and follow-up requirements must be considered. Furthermore, both sides of the telemedical support call need to take into account intramural possibilities for treatments and compliance of the patient, as well as individual preferences for treatment. To conduct a break-even analysis from the prison’s view, transparency about all costs and benefits must be provided [33]. With the emerging lack of specialists in Germany, the option for telemedical support in prisons as well as other facilities has advantages that should be considered in the health services for patients with limited access to regular medical facilities.

Textbox 1. **Benefits and limitations of teledermatology in the setting of teleconsultations for German prisons.**

**Benefits**

- Accessibility
- Transport and personnel deployment
- Expert exchange

**Limitations**

- Need for general inspection or biopsy
- Data provision
- Photo quality

## Abbreviations

KBV: Kassenaerztliche Bundesvereinigung
MBO-Ä: Muster-Berufsordnung für die in Deutschland tätigen Ärztinnen und Ärzte
